# Daily profile of COVID-19 infections in Europe – a biophysical perspective

**DOI:** 10.1007/s12551-025-01383-x

**Published:** 2025-12-18

**Authors:** Derek Marsh

**Affiliations:** Max-Planck Institute for Multidisciplinary Sciences (Formerly, Max-Planck-Institut für biophysikalische Chemie), 37070 Göttingen, Germany

**Keywords:** COVID-19, Exponential growth, Reproduction number R_0_, Variants of concern, Testing, Vaccination

## Abstract

Progression of the European COVID-19 pandemic is monitored using daily cases and associated deaths, reported in Italy, Germany and England. Weekly periodicity in reporting is filtered out with a moving average over a 7-day window. This reveals underlying stages of exponential growth and decay, and changes in response to preventative interventions. Exponential rate constants ${r}_{t}$, combined with different serial-interval distributions, yield estimates for the basic reproduction number R_0_ and instantaneous R_*t*_, and characterize the emergence of successive dominant viral variants. Rates of testing are discussed in detail, and corrected for. COVID-associated deaths are linked with daily cases, and fatality/case ratios (cfr) used to estimate the extent of under-reporting in the early stages. Reproduction numbers, R_0_ and R_*t*_, provide estimates of vaccine coverage required to reach population-level immunity, and subsequent modifications needed during the vaccination programme. Hence, we obtain a straightforward integrated description of the pandemic that is essentially biophysical.

**Graphical abstract:**

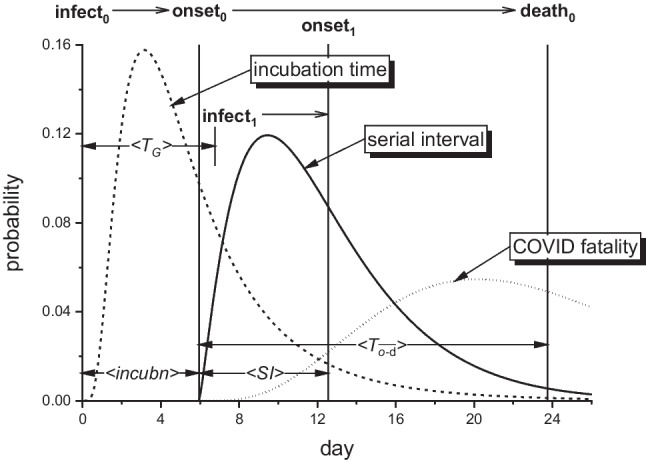

## Introduction

The pandemic caused by SARS-CoV-2 profoundly influenced the lives of us all, and the consequences are likely to occupy us for some time to come. Assessing the effectiveness of the control measures introduced, the impact of long COVID, and developing strategies for handling pandemics in the future, are among the major concerns. A retrospective review of COVID-19 incidence is therefore not without its value. This work began, of course, early in the pandemic. I write it from the point of view of a biophysicist, and inevitably of a potential infectee. Personally, it has helped me to appreciate better what Ian Smith told me over the years about his new work and achievements on founding the NRCC Institute of Biodiagnostics in Winnipeg. I like to imagine that there might be − at least weak − parallels with that heroic shift of fields, which he undertook.

Confirmed new cases of COVID-19 infection display a pronounced variation over the days of the week: the so-called “weekend effect”. We see this 7-day periodicity most clearly in data with systematically defined days of reporting. For example, the day of reporting a confirmed case to the local health authorities in Germany, or the day at which a specimen is taken for testing in Italy or in England. Here, I reveal the underlying progress of infection simply with a moving average over a 7-day window (Marsh 2025a). This lets me identify regions of exponential growth and decay from log-linear plots. Reproduction numbers R_0_ then follow from the exponential rate constants *r* − given auxiliary information on the distribution of generation times. R_0_ is the appropriate tool to follow the progression of the epidemic, to assess the efficiency of preventative interventions, and crucially to predict the level of vaccination coverage that provides population-wide protection. I use a similar approach with COVID-associated deaths. Connection with the profile of identified infections then lets us appreciate the likelihood of ensuing fatalities.

I apply this treatment to review COVID incidence in three representative countries of Western Europe. Italy was first to report a significant outbreak. England identified the first cases involving a new significant variant (Alpha), and was first to implement a national vaccination programme. Germany remains the most populous country, and home to the development of one of the most successful vaccines. The structure of the review is as follows: (i) Summary of mathematical background. (ii) Weekly modulation in reporting. (iii) Exponential waves of incidence compared in the three countries. (iv) Basic reproduction numbers, and timelines of the instantaneous R_*t*_. (v) COVID-variants of concern and their chain of interconversion. (vi) The contentious issue of testing rates and their influence on infection profiles. (vii) COVID-fatalities and infection profile. (viii) Prognoses and conclusion.

## Theoretical background

### Compartmental models and exponential growth

**Fig. 1:**
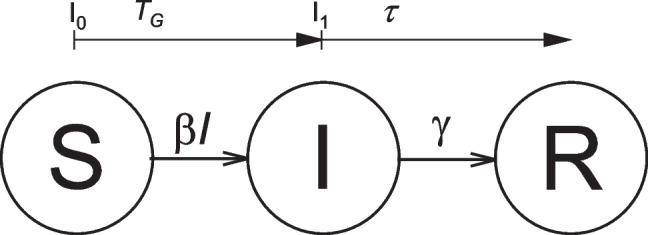
Susceptible-infectious-removed (SIR) 3-compartment model. S-to-I transitions, I_0_ to I_1_, take place after generation time ${T}_{G}$, at *per-capita* rate $\beta$; I-to-R transitions after infection lifetime $\tau$, at rate $\gamma$

For the basic susceptible-infectious-removed (SIR) model depicted in Fig. 1 (Kermack and McKendrick 1927), the rate equations for the fractional population (or concentration) of susceptible and removed (or recovered) compartments, $\left[S\right]=S/N$ and $\left[R\right]=R/N$, are:

1 $$d\left[S\right]/dt=-\beta\left[S\right]\left[I\right]$$

2 $$d[R]/dt=\gamma [I]$$

where $\left[I\right]=I/N$ is the fractional infectious population, β is the rate of transmission by an infectious individual, and γ is the recovery (or removal) rate of an infectious individual. The numbers of susceptible, infectious and removed individuals are *S*, *I* and *R*; and $N=S+I+R$ is the total population. The right-hand side of Eq. 2 is the recovery rate of the infectious population *I*. Therefore, the time for recovery from infection is distributed exponentially, with mean value $1/\gamma$ in this model (Hethcote 2000); more realistic distributions come later. The second-order rate constant for transmission *β* depends on the contact frequency of susceptibles with infectors multiplied by the probability of infection on contact, as in the law of mass action (de Jong et al. 1995; Hethcote 2000).

When the total population (*N*) remains fixed, the rate of increase in infectious population (*I*) is the difference between infection and recovery rates, Eqs. 1 and 2:

3 $$d[I]/dt=-d[S]/dt-d\left[R\right]/dt=(\beta \left[S\right]-\gamma )\left[I\right]$$

If the number of infections is low, relative to the population of susceptibles, [*S*] remains approximately constant over a limited period. According to Eq. 3, the infectious population (and hence daily fraction of new infections, $-d[S]/dt$ from Eq. 1) then grows exponentially with effective rate constant $r=\beta [S]-\gamma$. At the beginning of the epidemic, $[S_0]\approx{1}$  and the exponential rate constant is $r_0\;=\;\beta-\gamma$. Correspondingly, the number of new infections decreases exponentially when $[S]<\gamma /\beta\ (\equiv 1/{\mathrm{R}}_{0})$.

## Basic reproduction number, $R_0$  

The basic reproduction number ${\mathrm{R}}_{0}$ is the average number of new infections produced by a typical individual throughout their infectious lifetime, when the entire population is susceptible. We define a generation interval $\tau$ as the time from infection of an individual to infection of a secondary case by that individual (cf. Figure 1). Expressed *per capita*, the rate of transmission is the number per unit time $n\left(\tau \right)$, where $n\left(\tau \right).d\tau$ is the number of infections produced by an individual in time interval $\tau$ to $\tau +d\tau$ since becoming infected. The reproduction number is the sum over all $\tau$:

4 $${\mathrm{R}}_{0}=\underset{0}{\overset{\infty }{\int }}n\left(\tau \right).d\tau$$

Written in terms of transmission rate $n\left(\tau \right)$, the probability density function $g\left(\tau \right)$ for generation interval $\tau$ between primary and secondary infections is:

5 $$g\left(\tau \right)=n\left(\tau \right)/{\mathrm{R}}_{0}$$

where we use Eq. 4 for the normalizing denominator. The number of new infections at time *t* is the sum of all infections caused by individuals infected $\tau$ days ago, i.e., at times $t-\tau$. This results in the renewal equation:

6 $$C\left(t\right)=\underset{0}{\overset{\infty }{\int }}[S\left(t\right)]C\left(t-\tau \right)n\left(\tau \right).d\tau ={\mathrm{R}}_{0}[S\left(t\right)]\underset{0}{\overset{\infty }{\int }}C\left(t-\tau \right)g\left(\tau \right).d\tau$$

where *C*(*t*) is the daily count of new infections, and we use Eq. 5 for the right-hand side. Here, ${\mathrm{R}}_{0}\left[S\left(t\right)\right]$ is the reproduction number at time *t*, where ${\mathrm{R}}_{0}$ is the basic reproduction number at $t=0$ when $\left[S\left(0\right)\right]=1$.

We see from the renewal equation that the daily instantaneous reproduction number, ${\mathrm{R}}_{t}\equiv {\mathrm{R}}_{0}\left[S\left(t\right)\right]$, is the number of new infections ${C}_{t}$ at day *t*, divided by the total number of infectors causing these infections (Fraser 2007):

7 $${\mathrm{R}}_{t}=\frac{{C}_{t}}{{\sum }_{i=1}^{n}{C}_{t-{\tau }_{i}}{g}_{{\tau }_{i}}}$$

where ${\sum }_{i=1}^{n}{g}_{{\tau }_{i}}=1$, i.e., *n* is the number of days over which the probability density for the generation time $g(\tau )$ is discretized. Generation times ${\tau }_{i}$ are always positive. However, if we use serial intervals as proxy, ${\tau }_{i}$ becomes negative whenever infectiousness precedes symptoms. The instantaneous ${\mathrm{R}}_{t}$ defined in Eq. 7 gives the number of new infections produced by an individual infected at day *t*, if conditions remain those prevailing at *t* (Fraser 2007). It depends on the backwards-directed distribution of generation times, because it derives from the number of secondary infections produced at day *t*, cf. the denominator in Eq. 7 (see also Gostic et al. 2020).

A straightforward example of instantaneous reproduction number arises when the distribution is a delta function, $g\left(\tau \right)=\delta (\tau -{T}_{G})$. Equation 7 then becomes:

8 $${\mathrm{R}}_{\mathrm{t}}={C}_{t}/{C}_{t-{T}_{G}}$$

where ${T}_{G}$ is the single unique generation time. The instantaneous ${\mathrm{R}}_{t}$, for this case, is simply the ratio of daily new cases distanced ${T}_{G}$ days apart.

In regions where the rate of change in incidence varies exponentially, $C\left(t\right)={C}_{o}\mathrm{exp}(rt)$, the renewal equation (Eq. 6) becomes (Wallinga and Lipsitch 2007):

9 $$\frac{1}{{\mathrm{R}}_{0}}=\underset{0}{\overset{\infty }{\int }}{e}^{-r\tau }g\left(\tau \right).d\tau$$

Therefore, the inverse of the basic reproduction number $(1/{\mathrm{R}}_{0})$ is the Laplace transform of the generation-time probability density $g\left(\tau \right)$, with respect to the exponential rate constant *r* for infection. This is the demographers’ Lotka-Euler equation, where *r* is the Malthusian parameter.

In the SIR model, the distribution of infectious lifetimes is exponential: $g\left(\tau \right)=\gamma \mathrm{exp}(-\gamma \tau )$, when γ is fixed. For constant β this is also the distribution of generation times. Taking the Laplace transform, the reproduction number for an exponential distribution of generation times is:

10 $${\mathrm{R}}_{0}=1+r{\overline{T} }_{G}\space (=\beta /\gamma )$$

where ${\overline{T} }_{G}\equiv 1/\gamma$ is the mean generation time. However, the gamma distribution offers a more realistic probability density function for the infective period (Bi et al. 2020; Cereda et al. 2020):

11 $$g\left(\tau \right)=\frac{{(m\gamma )}^{m}}{\Gamma (m)}{\tau }^{m-1}{e}^{-m\gamma \tau }$$

where the mean is again ${\overline{T} }_{G}=1/\gamma$, and the standard deviation is $SD=1/\left(\gamma \sqrt{m}\right)$. This distribution is equivalent to *m* successive exponential stages each of duration $1/(m\gamma )$ (Lloyd 2001). From the Laplace transform, the reproduction number is:

12 $${\mathrm{R}}_{0}={(1+r{\overline{T} }_{G}/m)}^{m}$$

where we get *m* from the SD. Sometimes, the Gaussian distribution is used because this allows negative values of the serial interval, SI (see Ali et al. 2020). The reproduction number then becomes (Marsh 2025b):

13 $${\mathrm{R}}_{0}=\frac{1-\Phi \left(\left({\tau }_{m}-{\overline{T} }_{G}\right)/\sigma \right)}{1-\Phi \left(\left({\tau }_{m}-{\overline{T} }_{G}\right)/\sigma +\sigma r\right)}\mathrm{exp}\left({\overline{T} }_{G}r-\frac{1}{2}{\sigma }^{2}{r}^{2}\right)$$

Where $\sigma \equiv SD$ is the standard deviation; ${\tau }_{m}\le 0$ is the lower limit for the integrals given above, which we include to allow negative SIs; and $\Phi \left(x^{\prime}\right)={\int }_{-\infty }^{x^{\prime}}\mathrm{exp}\left(-\frac{1}{2}{x}^{2}\right).dx/\sqrt{2\pi }$ is the cumulative distribution function up to $x=x^{\prime}$, for a normal distribution. When using SIs as proxy for ${T}_{G}$, in principle the lower limit extends to ${\tau }_{m}\to -\infty$, which yields $\Phi \left(-\infty \right)=0$ in Eq. 13 and hence:

14 $${\mathrm{R}}_{0}=\mathrm{exp}(r{\overline{T} }_{G}-\frac{1}{2}{r}^{2}{\sigma }^{2})$$

This is the result usually quoted for a Gaussian distribution (e.g., Wallinga and Lipsitch 2007; Du et al. 2020; Ganyani et al. 2020), although it should be used only with very narrow distributions, i.e., for $\sigma \to 0$ (see Marsh 2025b). For a delta-function distribution, Eq. 9 gives:

15 $${\mathrm{R}}_{0}=\mathrm{exp}(r{T}_{G})$$

which is a special case of Eq. 8 for the exponential regime.

**Fig. 2:**
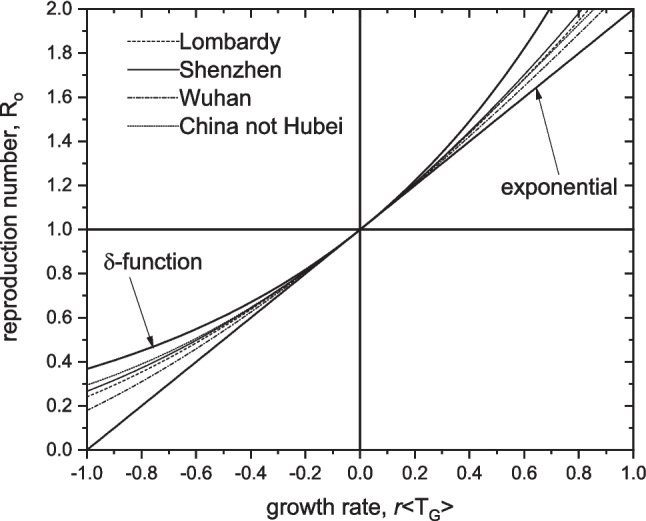
Dependence of the reproduction number ${\mathrm{R}}_{0}$ on rate constant *r* for exponential growth scaled by the mean generation time $\langle {T}_{G}\rangle$ for different distributions of ${T}_{G}$, according to Eqs. 10, 12, 14 and 15. Data for gamma distribution from Cereda et al. (2020), dashed line; Bi et al. (2020), solid line; Li et al. (2020), dashed-and-dotted line; and for Gaussian distribution from Ali et al. (2020), Eq. 14 dotted line. Outer solid lines are for δ-function and exponential distributions, as indicated

Figure 2 shows the dependence of reproduction number ${\mathrm{R}}_{0}$ on the exponential rate constant *r* that we predict from the above models. For fixed mean generation time, an exponential distribution gives the lowest values of ${\mathrm{R}}_{0}$, and the delta-function defines the upper limit. As ${\mathrm{R}}_{0}$ approaches one, differences between the models become unimportant (Wallinga and Lipsitch 2007).

### Outcomes of the epidemic

We can deduce final outcomes by taking the limit $t\to \infty$ when solving Eqs. 1, 3 for the SIR model. From Eqs. 1, 3 we get d[*I*]/d[*S*], which integrates to:

16 $$\left[I\right]={[I]}_{o}+{[S]}_{o}-\left[S\right]+(\gamma /\beta )\mathrm{ln}([S]/{[S]}_{o})$$

where subscripts refer to $t=0$. Taking initial conditions ${[I]}_{o}\cong 0$, i.e., ${[S]}_{o}\cong 1$, and final condition ${[I]}_{\infty }=0$ as $t\to \infty$, we get the total fraction of infections by the end of the outbreak, $\rho \equiv 1-{[S]}_{\infty }$, from:

17 $$1-\rho =\mathrm{exp}(-{\mathrm{R}}_{0}\rho )$$

where ${\mathrm{R}}_{0}=\beta /\gamma$ in the SIR model. This is the final-size equation. At this point, we reach herd immunity. Earlier in the outbreak, the infectious population attains a maximum ${[I]}_{max}$, before decaying to zero as $t\to \infty$. Without preventative interventions, the maximum occurs when d[*I*]/d*t* = 0, for which $[S]=\gamma /\beta =1/{\mathrm{R}}_{0}$ (see Eq. 3). Using Eq. 16, we then get:

18 $${\left[I\right]}_{max}=1-\left(1+\mathrm{ln}\left({\mathrm{R}}_{0}\right)\right)/{\mathrm{R}}_{0}$$

with the initial conditions introduced previously. Note that the final size deduced from Eq. 17 applies more generally than the SIR model, amongst other things including both non-zero latency and general distributions of infective period (Ma and Earn 2006).

Alternatively, we can replace the deterministic SIR model by using probabilistic descriptions of the initial outbreak as a branching process. For a gamma distribution, the probability $\Pi$ of developing a major outbreak (i.e., exponential growth) is the solution to the balance equation (Anderson and Watson 1980; Britton and Lindenstrand 2009):

19 $$1-\Pi ={\left(1+\Pi {\mathrm{R}}_{0}/m\right)}^{-m}$$

where *m* is as defined in Eq. 11, and ${\mathrm{R}}_{0}$ is given by Eq. 12. The gamma distribution better approximates the serial intervals found with COVID-19 infections (Li et al. 2020; Bi et al. 2020; Cereda et al. 2020). Note that as $m\to \infty$, Eq. 19 tends in the limit to $1-\Pi =\mathrm{exp}(-{\mathrm{R}}_{0}\Pi )$, corresponding to fixed infectivity (up to $\tau =1/\gamma$, see Eq. 11). On the other hand, $m=1$ (i.e., an exponential distribution—see Eq. 11) yields $\Pi =1-1/{\mathrm{R}}_{0}$. The mean final size of the outbreak is $\rho$ conditional on the occurrence of a major outbreak, which for large populations ($N\to \infty$) is close to the solution of Eq. 17, as expected for a central limit (Britton and Lindenstrand 2009; Anderson and Watson 1980).

## Daily cases of COVID-19

### Weekly modulation of reporting

**Fig. 3:**
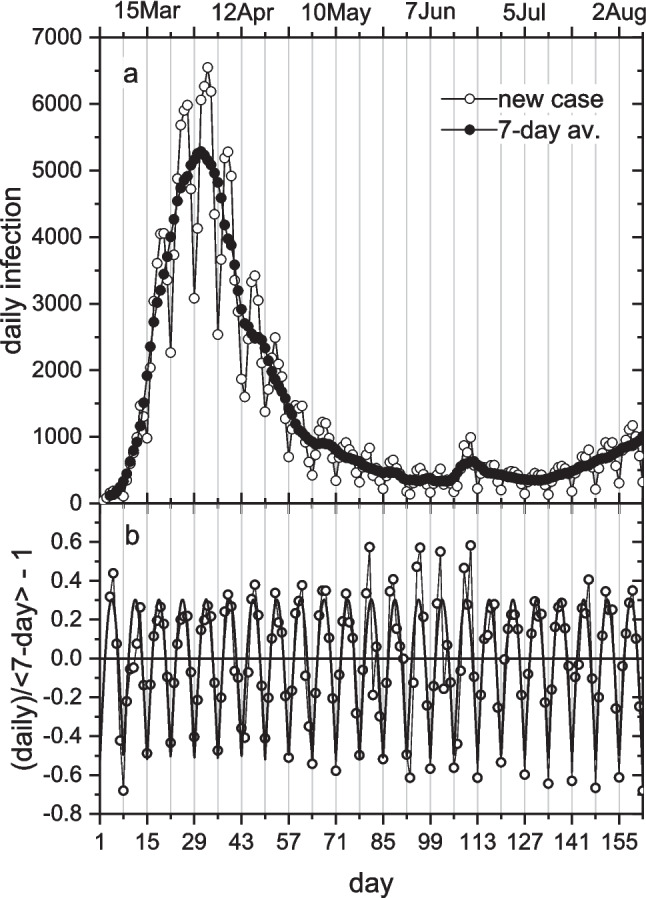
**a**) Daily number of new COVID-19 cases in Germany according to reporting date (open circles), and 7-day moving average (solid circles). Data from Robert-Koch Institute website (RKI 2021a). **b**) Weekly modulation factor, difference between daily cases and 7-day average, normalized to the latter (circles). Nonlinear least-squares fit of absolute sine function (heavy line). Day-1 is 1 Mar 2020

Open circles in Fig. 3a give the new COVID-19 cases reported each day to the local health authorities in Germany. Solid circles are averages over a 7-day window centred on each day plotted. Days are numbered consecutively, starting from 1 Mar 2020 as day-1; the time span shown covers the first wave of infection and the following summer trough. We see a very strong weekly periodicity in incidence, when based on the official day of reporting, which the moving average filters out. The solid circles then reveal the underlying progress of the epidemic. Therefore, we concentrate henceforth on 7-day averaged incidences.

Figure 3b shows the amplitude of the weekly modulation that we get by dividing the original data by the 7-day average. This is well fitted, up to day-297, by an absolute sine function, $\sim |\mathrm{sin}(\pi (t-{t}_{o} )/wk)|$, used originally by Dehning et al. (2020a). After day-297, the modulation changes, reflecting different reporting patterns: the amplitude increases, and the maximum shifts from midweek towards the beginning of the week. Beyond day-733, the modulation increases abruptly coincident with a large drop in rate of testing at the end of the 2021–2022 winter wave (see later, and Marsh 2025a). Weekly modulation of daily case numbers also occurs in incidence data from Italy and England. Quantitative differences appear between countries because of different circumstances and reporting protocols. The amplitude of 7-day modulation decreases in the order Germany > Italy > England.

### Rate of daily cases in Italy, Germany and England

**Fig. 4:**
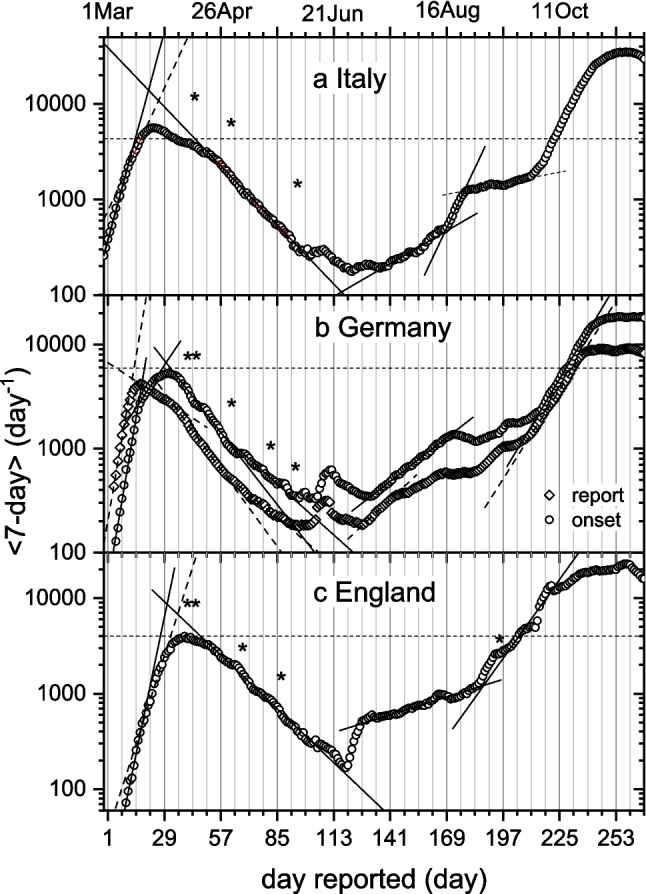
Daily number of new cases in: **a**) Italy, 7-day moving average according to reporting date; **b**) Germany, 7-day averages according to reporting date (circles) or onset of symptoms (diamonds); **c**) England, 7-day average according to publishing date. Asterisks indicate public holidays (day-1 = 1 Mar 2020); horizontal line: weekly incidence, 50 per 100,000 inhabitants; *y*-axis is logarithmic. Data from: a) Dipartimento-della-Protezione-Civile website (DPC 2021); b) RKI (2021a); c) UK-Government website (UK-Gov 2021a)

Figures 4a, b, and c compare the 7-day average daily cases for Italy, Germany and England, respectively, on a log scale. The time period covered includes the primary wave, summer trough, annual holidays, and subsequent autumn/winter wave, all in 2020. Horizontal lines correspond to a weekly incidence of 50 per 100,000 inhabitants. (This initial regulatory metric was soon exceeded, and ultimately reached peaks of ca. 2,000 − 2,200.) Fig. 4b for Germany includes the timeline for the onset of symptoms, which precedes that for reporting by ca. 5 days. Interestingly, the shapes of the profiles for the initial outbreak in Italy and in England resemble more that for the onset data from Germany than that for the reporting date. Italy was first in western Europe to report an outbreak, and we see from Fig. 4a that the first wave begins before those for reporting in Germany and in England. The outbreak was already underway before testing in England became well developed. We see this from the shorter delay between incidence and fatality profiles that comes in a later section. Another feature of the England data (Fig. 4c) is the jump at 2 Jul (day-124) caused by changing data source such that the new figures do not compare directly with the old figures (UK-Gov. 2025). The jump occurs close to an abrupt inclusion of Pillar-2 testing on 14 Jul (day-136), which was introduced at the time. For Germany (Fig. 4b), a local singularity in basal level at around day-107 (Jun 15) originates from severe outbreaks of infection in the meat-processing industry and centres of high-density housing. It is prominent because it occurs in the summer trough.

Public holidays are marked with asterisks in Fig. 4. We can expect dips in reporting here, followed by filling up with the delayed cases, analogous to the weekend periodicity of Fig. 3. This appears in the profile for reporting in Germany (Fig. 4b), where short plateau regions, following the Easter period (day-43) and 1-May holiday (day-62), are preceded by sharper decreases. Such irregularities are absent for onset-of-symptoms, suggesting that the short public holidays had a relatively minor effect on true incidence rates under preventative measures in place at the time. The correlation is less marked for the two other countries, but with some clear indications in England (Fig. 4c). Later, very clear local discontinuities occur at the Christmas/New Year period (day-300/−307) (see Figs. 6, 8 and 10 below).

**Fig. 5:**
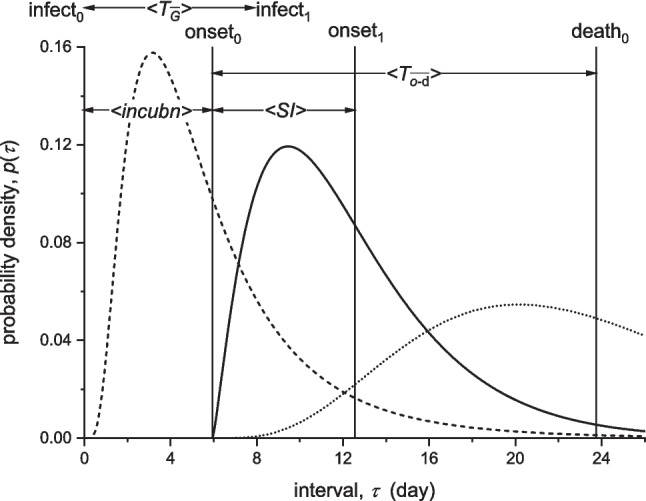
Probability densities for time delays in transmission of COVID-19 infection. Primary case is infected at $\tau =0$, with mean incubation time to onset-of-symptoms $\langle incubn\rangle =6.0$ days (dashed line, lognormal distribution; Bi et al. 2020). Onset-of-symptoms of secondary infected case follows at mean serial interval $\langle SI\rangle =6.3$ days (solid line, gamma distribution; Bi et al. 2020). COVID-associated deaths occur at mean time from onset-of-symptoms $\langle {T}_{o-d}\rangle =17.8$ days (dotted line, gamma distribution; Verity et al. 2020). Heavy vertical lines are means of the corresponding distributions. $\langle {T}_{G}\rangle$ indicates the generation time schematically

The plots in Figs. 4a, b, c reveal linear sections of the profiles that represent regions of exponential growth or decay in transmission of infection with rate constants ${r}_{0}$ to ${r}_{5}$ given in Table 1. The fast initial rates ${r}_{0}$, before any interventions, correspond to doubling of infections every 2.6, 2.9 and 3.9 days in Germany, England and Italy, respectively. It is not straightforward to correlate subsequent decreases in exponential rates of incidence with the timing of measures introduced to control the pandemic. Firstly, interventions were very closely spaced in the first phase of the epidemic, and secondly uncertainties inevitably arise from delays in reporting. Symptoms onset lies closer to the date of original infection than does reporting. It depends on the distribution of incubation times, which has a mean value of 5–6 days (Lau et al. 2021; Lauer et al. 2020; and see Fig. 5 later). We concentrate first on onset-of-symptoms data, given by open diamonds in Fig. 4b for Germany. The initial and fastest region of growth extends to day-10/11. Beyond this, the growth rate slows progressively, falling to zero at maximum incidence on day-16/17. Then follows an exponential decrease that changes to a two-fold faster rate (${r}_{2}$) at around day-32/33. In Germany, mass gatherings were banned from 8 Mar (day-8); schools and day-care centres closed from 13 Mar (day-13); public spaces, including non-essential shops, bars and restaurants, and entertainment venues, were closed from 16 Mar (day-16); and general lockdown with advice to stay at home came on 17 Mar (day-17) (ECDC 2021). Allowing for incubation time, the initial decrease in exponential rate precedes the first restrictions, and likely results from growing public awareness. Maximum daily incidence and initial decay follow closely on the first two interventions, whereas the doubling in decay rate comes well after closure of public spaces and general lockdown. Possibly, seasonality contributes synergistically with the descent into the summer trough. The ${r}_{2}$-decay rate slows down at around day-59/60. This follows the first easing of restrictions: opening of small shops, bookstores and restricted areas in larger shops in mid-April (day-46/47), and precedes complete lifting of lockdown on 4 May (day-65). Change points in incidence timeline that correspond with non-pharmaceutical interventions already were discussed early in the pandemic (e.g., Dehning et al. 2020a,b; Flaxman et al. 2020).

**Table 1:** Initial rate constant for infection (and COVID-associated deaths) ${r}_{0}$, and subsequent decrease ${r}_{2}$ with following increases ${r}_{4}$ and ${r}_{5}$, in Germany, Italy and England ^a^

country	${r}_{0}$ (day^−1^)	${r}_{2}$ (day^−1^)	${r}_{4}$ (day^−1^)	${r}_{5}$ (day^−1^)
*Incidence:*				
Germany	0.264 ± 0.007^b^ [5,16]^c^	− 0.058 [36,66]	0.035 [132,172]	0.074 [217,242]
Italy	0.179 ± 0.003 [−1,11]	− 0.051 [50,91]	0.103 [170,178]	0.094 [221,238]
England	0.236 ± 0.006 [8,19]	− 0.046 [50,107]	0.015 [134,163]	0.068 [201,214]
*Deaths:*				
Germany	0.267 ± 0.014 [11,18]	− 0.056 [53,102]	-	0.079 [214,249]
Italy	0.268 ± 0.008 [0,11]	− 0.041 [50,91]	-	0.094 [226,249]
England^d^	0.230 ± 0.004 [15,23]	− 0.040 [51,107]	-	0.056 [184,237]

^a^From log-linear regressions in Figs. 4, 9

^b^The uncertainties (±) are standard errors in the slope SE = SD/√(∑(*t*_*i*_-$\overline{t}$)^2^), where SD and *t*_*i*_ are standard deviation of the fit and day-numbers, respectively. SE for ${r}_{2}$, ${r}_{4}$, ${r}_{5}$ is less than for ${r}_{0}$ (typically ≅ 0.001 day^−1^)

^c^Square brackets: fitting range in day-number

^d^Deaths up to 28 days after diagnosis (UK-Gov. 2022b)

Data for reporting dates in Germany (open circles in Fig. 4b) mostly track those for symptoms onset. Transitions already highlighted for the latter appear 3 − 5 days later in reporting, although this is not just a constant delay. Shape differences between the first peaks show that the distribution in reporting delays can distort the incidence profile. Growth rates ${r}_{t}$ for onset of symptoms are similar to those given in Table 1 for reporting, except that the exponential changes occur earlier. From the summer trough onwards in Fig. 4b, reporting data clearly parallels that for symptoms onset. The incidence rate for symptoms onset begins to increase at day-126 (4 Jul; that for reporting date four days later). This coincides with the start of German school holidays, which are staggered between the different federal states and extend from day-114 to day-196. The steeper increase for Italy in August similarly corresponds with the annual summer holidays (Fig. 4a). The later sharp rise of daily incidence beginning in October (day-215; ${r}_{5}$-range in Table 1) is the seasonal increase at the onset of winter common to all three countries. Seasonality was evident already towards the beginning of the pandemic in Europe from the shifted profiles of daily incidence in the southern hemisphere, e.g., for Australia and South Africa (ECDC 2020).

## Basic reproductive number, $R_0$  

The basic reproduction number is the average number of infections produced on introducing a single infectious individual into a homogeneous population of susceptibles. It is important for assessing the strength of interventions and the extent of vaccination needed to halt the epidemic. The epidemic grows when ${\mathrm{R}}_{0}>1$, and declines when ${\mathrm{R}}_{0}<1$.

We get ${\mathrm{R}}_{0}$ from values of the initial exponential growth rate, ${r}_{0}$, in Table 1 by using Eq. 9 which we obtained from the renewal equation. To do this we need the distribution $g\left(\tau \right)$ of generation times ${\tau =T}_{G}$, which leads to Eqs. 10, 12 − 15 (for the various distributions) that relate ${\mathrm{R}}_{0}$ to ${r}_{0}$ and to the mean generation time $\overline{T }_{G}$. Because $\overline{T }_{G}$ is not directly accessible, we use serial intervals (SI), which are accessible to observation, instead. These are the difference in observable onset of symptoms for primary and secondary infections, as opposed to difference in times of infection that defines ${T}_{G}$ (see Fig. 5). If incubation times (i.e., time from infection to symptoms onset) are the same for primary and secondary cases, values of SI equal those of the generation period. A spread in incubation times increases that for SI relative to ${T}_{G}$ (Ganyani et al. 2020; Lehtinen et al. 2021). For ${\mathrm{R}}_{0}$, we need serial intervals from early stages of the epidemic, because they decrease as protective measures are applied (Ali et al. 2020; Du et al. 2020).

Within Europe, the mean SI for close contacts of positive cases is 6.6 days with standard deviation SD = 4.8 days, deduced using a gamma distribution from the early phase of the outbreak in Lombardy, Italy (Cereda et al. 2020). For early stages of the well-defined outbreak in Shenzhen, China, the mean SI is 6.3 days (SD = 4.2 days; gamma distribution) (Bi et al. 2020). Early data for Wuhan, China give mean SI = 7.5 days (SD = 3.4 days; gamma distribution) (Li et al. 2020). In the initial, pre-peak period of infection, the mean SI for mainland China excepting Hubei Province, is 7.8 days (SD = 5.2 days; Gaussian distribution), which decreases subsequently as preventative interventions are undertaken (Ali et al. 2020). Over the entire range, from pre- to post-peak, the mean SI drops to 5.1 days (SD = 5.3 days), which is comparable to reports that assume a single distribution for all stages.

For gamma distributions of SI (Eq. 12), the basic reproduction number is ${\mathrm{R}}_{0}\cong$ 3.4, 3.1 and 2.5 for Germany, England and Italy, respectively, when using SI-data from Lombardy (Cereda et al. 2020) and Shenzhen (Bi et al. 2020); and ${\mathrm{R}}_{0}\cong$ 5.3, 4.5 and 3.3 from the early Wuhan data where the SD is smaller (Li et al. 2020). Correspondingly, we get ${\mathrm{R}}_{0}\cong$ 4.4, 4.0 and 3.2 for Germany, England and Italy, respectively, using the Gaussian distribution (Eq. 13 and ${\tau }_{m}=-2$ days) with the pre-peak data for mainland China outside Hubei province (Ali et al. 2020); and ${\mathrm{R}}_{0}\cong 2.1-1.9$ for the entire dataset (${\tau }_{m}=-5$ days). Note that the frequently used Eq. 14 yields considerably lower values (e.g., Marsh 2025a). Here, we choose ${\tau }_{m}$ to match discrete R_*t*_-calculations from Eq. 7 with Gaussian probability density.

In contrast to these data-informed values, Eq. 15 for a delta-function gives us the upper limit, relative to distributions with non-vanishing dispersion and the same mean ${T}_{G}$ (see Fig. 2; Wallinga and Lipsitch 2007). Taking ${T}_{G}\cong$ 7 days as representative (cf. mean SI-values above), we get: ${\mathrm{R}}_{0}\cong$ 6.3, 5.2 and 3.5 as the upper limit for Germany, England and Italy, respectively, well above any foregoing estimate. The delta-function distribution is a unique case. Realistically, it seems more appropriate to a sharp peak in infectivity, i.e., in $\beta (\tau )$, than to a single unvarying contact time ${T}_{G}$ (see Fig. 5). For a gamma distribution (Eq. 11), the peak in infectivity occurs at infectious lifetime: ${\tau }_{max}=(1-1/m)/\gamma$, which is much smaller than 7 days.

For comparison, Flaxman et al. (2020) estimate ${\mathrm{R}}_{0}=3.8$ [CI: 2.4–5.6] from COVID mortality data when averaged over 11 European countries. For Germany, an der Heiden and Hamouda (2020) using Eq. 8 with ${T}_{G}=4$ days, and Dehning et al. (2020a) using the SIR model, effectively with a recovery time of 8 days, both estimate ${\mathrm{R}}_{0}=3.4$. Differing somewhat, Linka et al. (2020a) estimate ${\mathrm{R}}_{0}\cong 6.3\pm 0.6$ for Germany, from a susceptible-exposed-infectious-removed (SEIR) model with latent and infectious periods of 2.5 and 6.5 days, respectively. This high value comes from the longer recovery time and from using the SEIR model.

### Instantaneous reproduction number, R_t_

**Fig. 6:**
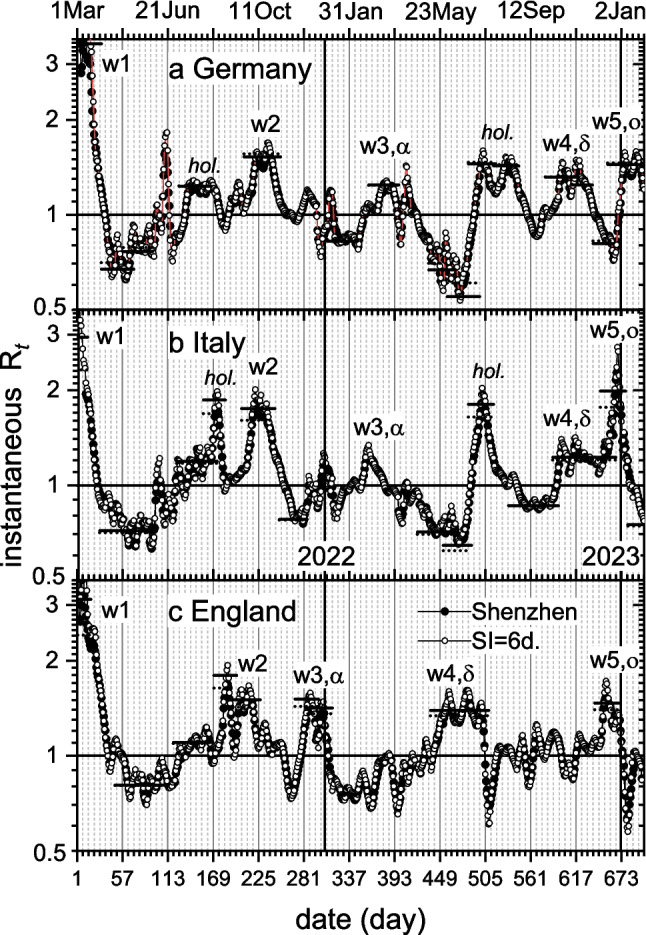
Instantaneous reproduction number R_*t*_ deduced from 7-day averaged daily incidences for: **a**) Germany, **b**) Italy, **c**) England. Reporting date for Germany and Italy, and sample date for England. Solid circles for gamma-distributed SIs (Bi et al. 2020; Eq. 7); open circles for delta distribution (Eq. 8) with ${T}_{G}=6$ days. Horizontal lines are R_*t*_ from exponential incidence rates *r*_*t*_ (Eq. 9). Solid lines: Eq. 12 (gamma-distributed SI); dotted lines: Eq. 15 (${T}_{G}=6$ d). Infection waves (w1, w2, …), including those associated with COVID-variants (w3,α; w4,δ; …) are labelled. *hol.* denotes summer holidays. Day-1 is 1 Mar 2020; *y*-axis is logarithmic

We follow variations in reproduction numbers from the 7-day averaged daily incidences *C*_*t*_, by using Eqs. 7 and 8. Figure 6 shows the time-progression of the instantaneous reproduction number ${\mathrm{R}}_{t}$ in Germany, Italy and England. Solid circles are for a gamma distribution of SIs (Bi et al. 2020; Eq. 7); and open circles are from the delta distribution (Eq. 8), with fixed ${T}_{G}=6$ days that is close to the mean SI of Bi et al. (2020). Time dependence is more erratic for the open circles because they depend on only two values of *C*_*t*_, whereas convolution with the SI distribution smooths that for the solid circles. The *y*-axis of Fig. 6 is logarithmic, but this is simply to give more prominence to R_*t*_-peaks (and troughs) following the first R_0_-maximum.

**Fig. 7:**
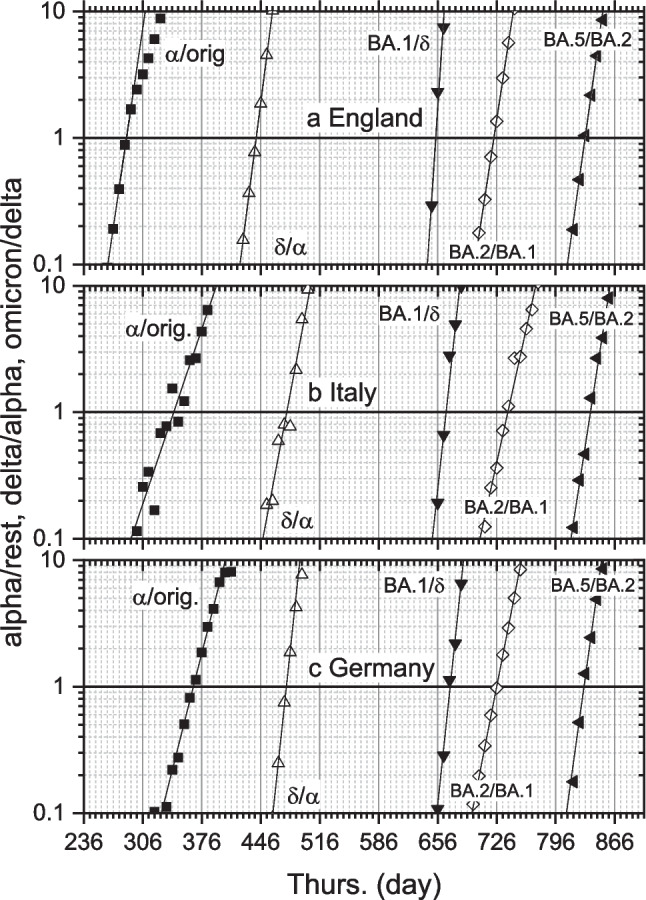
SARS-CoV-2 variants of concern, 2021–2022: original, Alpha (B.1.1.7), Delta (B.1.617.2), Omicron (B.1.1.529 ≡ BA.1), and Omicron-variants BA.2 and BA.5. Ratio of weekly cases for newly dominating mutants relative to the previous one, in changeover regions: Alpha/original (solid squares), Delta/Alpha (open triangles), BA.1/Delta (solid inverted triangles), BA.2/BA.1 (open diamonds), BA.5/BA.2 (solid left triangles). **a**) England (UK-Gov. 2021b), **b**) Italy (ECDC 2023a), **c**) Germany (RKI 2021b; 2023a). *y*-axis is logarithmic; straight lines: linear regressions in common changeover ranges; *x*-axis: each weekly point is assigned to a Thursday

Two major features characterize the progressions in Fig. 6: (i) Maxima and minima in ${\mathrm{R}}_{t}$ correspond, respectively, to the largest positive and negative rates of incidence, *r*_*t*_; (ii) Values cross ${\mathrm{R}}_{t}=1$ where incidence is maximum or minimum. In each panel, the first maximum, at the rise of wave w1, is sharp and overflows the plotting limits. However, 7-day averages about the peak centres agree with the solid horizontal bars, which are ${\mathrm{R}}_{0}$-values deduced already above from Eq. 9, for England and Germany with SI-data from Bi et al. (2020). Subsequent extrema agree more directly with the heavy horizontal bars. These ${\mathrm{R}}_{t}$-values are deduced from exponential rates *r*_*t*_ by using Eqs. 12 (solid) and 15 (dotted) that correspond to the solid and open circles, respectively. Solid and dotted bars are mostly close together because they correspond to similar mean SIs, and lie reasonably close to ${\mathrm{R}}_{t}=1$ (cf. Figure 2). They are labelled according to the current incidence wave and dominant COVID-variant. We see major differences mainly between England and the other two countries. These are associated with the earlier appearance of the Alpha and Delta variants in England (see Fig. 7 later). Artefacts associated with short public holidays are accentuated in the ${\mathrm{R}}_{t}$-plots relative to incidence plots, e.g., at Christmas/New Year. After the first wave, all values stay below ${\mathrm{R}}_{t}=2$, but one should caution against detailed quantitative comparisons between countries. Definitions, protocols and procedures can differ according to country. Variabilities include those in: stringency of diagnosis (symptoms, antigen tests, or RT-PCR), under reporting, rates of testing, preventative interventions, vaccination strategies, and notoriously in what qualifies as a COVID-associated death. Subsequent maxima in ${\mathrm{R}}_{t}$, beyond the range of Fig. 6, correspond to appearance of various dominant Omicron variant sublines BA.2, BA.5, BF.7, BQ.1 and XBB1.5, in order (cf. Marsh 2025a, and Fig. 10 given at the end).

We turn briefly now to the rate constant *β* for transmission of infection (Eq. 1). The daily rate of transmission in Eq. 4 is related to *β* by: $n\left(\tau \right)=\beta {P}_{S}(\tau )$, where the survival probability for infection ${P}_{S}\left(\tau \right)$ integrates to give the mean lifetime. Hence, from Eq. 4:

20 $${\mathrm{R}}_{0}=\beta \underset{0}{\overset{\infty }{\int }}{P}_{S}\left(\tau \right).d\tau =\beta {\overline{T} }_{G}$$

where ${\overline{T} }_{G}$ is the mean infectious lifetime, which holds good for general distributions of lifetime. Using values of ${\mathrm{R}}_{0}$ deduced with the gamma distribution of SIs from Bi et al. (2020), the transmission rate constant becomes: $\beta =$ 0.54, 0.49 and 0.40 day^−1^ for Germany, England and Italy, respectively. For comparison in early stages of the outbreak in Germany, Dehning et al. (2020a) derive $\beta =0.41$ [0.32,0.51] day^−1^ from Bayesian inference with the SIR model and an informative prior of $\gamma =0.12$ day^−1^.

## New CoV-2 variants

As the pandemic progressed, SARS-CoV-2 variants of concern evolved that were more infective, or better able to evade the immune system. These therefore came to replace previous variants. Figures 7a, b, c give the ratio of positive cases for each new variant, relative to the previous one. Numbers come from weekly random sampling of positive cases with fully determined genomic sequences. The *y*-axis is logarithmic. First comes the Alpha variant, relative to the original strain, followed by Delta, then Omicron (BA.1), and finally new Omicron variants BA.2 and BA.5. Change over between variants occurs when the ratios are unity. The Alpha variant comes to dominate in England before Italy and Germany, and likewise for Delta. Omicron comes to dominate over Delta in a very short time interval, especially in England. This testifies to the extremely high infectivity of the original Omicron variant.

Incidences of both old and new variants are close to exponential in the crossover region (data not shown, but see Marsh 2025a). Therefore, the exponential dependence of the variant ratios seen in log-linear plots of Figs. 7a, b and c gives the difference $\Delta r={r}_{1}-{r}_{2}$ in exponential rates between old and new variants. We can relate these differences to corresponding effective reproduction numbers, ${\mathrm{R}}_{t,1}$ and ${\mathrm{R}}_{t,2}$, by using either of two simple SI-distributions. For an exponential SI-distribution (Eq. 10), we get the difference: ${\mathrm{R}}_{t,1}-{\mathrm{R}}_{t,2}$. Alternatively, for a delta-function SI-distribution (Eq. 15), we get the ratio: ${\mathrm{R}}_{t,1}/{\mathrm{R}}_{t,2}$. Table 2 lists these ${\mathrm{R}}_{t}$-parameters for the interchanging variants. We can use either of the two combinations as a measure for the increased infectivity *β* of the new variant. The results in Table 2 are based on a mean SI of 6.3 days from the data of Bi et al. (2020). For comparison, directly determined exponential rates with the gamma distribution of SI from Bi et al. (2020), give: ${\mathrm{R}}_{t,1}=1.20\pm 0.02$ and ${\mathrm{R}}_{t,2}=0.82\pm 0.02$ for Alpha and original variant at crossover, in Germany (Marsh 2025a). Because these separate values are reasonably close to one, they agree rather well with the combinations in Table 2 although they correspond to different models (see Fig. 2). In general, we cannot combine the ratios and differences in Table 2 to deduce individual ${\mathrm{R}}_{t}$ s, because they refer to incompatible models. However, if the resulting values of ${\mathrm{R}}_{t}$ are close to one, where Fig. 2 shows us that the particular model is unimportant, we have a simple and useful method to estimate the individual reproduction numbers for each variant.

**Table 2:** Effective reproduction numbers, R_t,1_ and R_t,2_, at changeover between dominant COVID-variants, 1 and 2^a^

variants	Germany		Italy		England	
	R_t,1_/R_t,2_^b^	R_t,1_-R_t,2_^ c^	R_t,1_/R_t,2_	R_t,1_-R_t,2_	R_t,1_/R_t,2_	R_t,1_-R_t,2_
α/orig	1.46 ± 0.03	0.38 ± 0.02	1.33 ± 0.03	0.29 ± 0.02	1.92 ± 0.08	0.65 ± 0.04
δ/α	2.48 ± 0.12	0.91 ± 0.05	1.67 ± 0.08	0.51 ± 0.05	2.12 ± 0.03	0.75 ± 0.01
BA.1/δ	2.50 ± 0.46	0.92 ± 0.18	2.44 ± 0.23	0.89 ± 0.10	4.31 ± 1.00	1.46 ± 0.23
BA.2/BA.1	1.64 ± 0.04	0.49 ± 0.03	1.55 ± 0.03	0.44 ± 0.02	1.88 ± 0.02	0.63 ± 0.01
BA.5/BA.2	2.00 ± 0.12	0.69 ± 0.06	1.87 ± 0.05	0.63 ± 0.03	1.96 ± 0.02	0.68 ± 0.01

^a^From difference in exponential rate constants, Δ*r,* for infection (see Fig. 7)

^b^From Eq. 15, with ${T}_{G}=6.3$ days (Bi et al. 2020)

^c^From Eq. 10, with ${\overline{T} }_{G}=6.3$ days (Bi et al. 2020)

Park et al. (2022) make a distinction in mechanism of infection enhancement between increases in speed given by $\Delta r$ and in strength given by ${\mathrm{R}}_{t,1}/{\mathrm{R}}_{t,2}$. The former we get directly from Fig. 7, and the latter depends additionally on choice of generation time (or SI) distribution. This approach helps us in assessing preventative interventions, if they fall predominantly into one of these two modes. The authors propose that social distancing or vaccination as constant-strength interventions would reduce transmission rate by a fixed proportion, whereas contact tracing ideally could isolate infectives at constant rate.

If recovery times depend more on the individuals infected and their local situation than on the particular viral variant, mean infectious lifetimes ${\overline{T} }_{G}$ might not vary greatly between variants. Equation 20 above then implies that the ${\mathrm{R}}_{t}$-ratios in Table 2 correspond directly to ratios in infectivity *β* of the variants, irrespective of SI-distribution. In fact, ratios derived for Germany by using explicitly the gamma distribution of Bi et al. (2020), lie very close to the ${\mathrm{R}}_{t}$-ratios in Table 2 that we base on a delta distribution with fixed ${T}_{G}$. For Alpha relative to the original variant: ${\beta }_{\alpha }/{\beta }_{\mathrm{orig}}$= 147 ± 5%; for Delta relative to Alpha: $\beta_\delta/\beta_\alpha$ = 242 ± 29%; for Omicron (BA.1) relative to Delta: ${\beta }_{o}/{\beta }_{\updelta }$ = 249 ± 39%; and for the Omicron subvariants: ${\beta }_{\mathrm{BA}.2}/{\beta }_{\mathrm{BA}.1}$ = 165 ± 8%, ${\beta }_{\mathrm{BA}.5}/{\beta }_{\mathrm{BA}.2}$ = 204 ± 13% (Marsh 2025a). However, reproduction numbers will change if generation-time distributions differ between variants. A statistical model for household transmission data in the UK reveals a decrease in ${\overline{T} }_{G}$ from 5.5 days [CI: 4.7,6.5] for the Alpha variant to 4.7 days [CI: 4.1,5.6] for the Delta variant (Hart et al. 2022). Thus, using the method above without correction somewhat overestimates the  $\mathrm{R}_{t,\delta}/\mathrm{R}_{t,\alpha}$ ratio. Using Eq. 20 for an estimate, the corrected transmissivity ratio reduces to approximately $\beta_\delta/\beta_\alpha$ = 207 ± 25%, with corresponding reduction in demands on vaccination (see *Prognoses* Section, later).

For comparison with the ${\mathrm{R}}_{t}$-ratios here in Table 2, Davies et al. (2021) estimate ${\mathrm{R}}_{Alpha}/{\mathrm{R}}_{other}$ = 143 − 190% (CI: 130–230%) for Alpha in England. The odds for transmission of Delta relative to Alpha is 1.70:1 (95% CI 1.48 − 1.95) from statistical estimates for household clusters in England (Allen et al. 2022). Additionally, overall transmissibility of Delta is estimated as 2.1 times that of Alpha (95% CI 1.3 − 3.3) for UK households (Hart et al. 2022). These values are similar to the estimates made here for Germany.

We should remember that reproduction numbers of new variants here are those prevailing at the time of changeover between dominant variants. These conditions are not necessarily the same at different changeover points. Insofar, however, as increased transmissivity is more a property of the viral variant than of the host and its environment, we can use data from adjacent changeovers to give us some idea of progressive change in transmissivity along the chain of variants. Even so, an idealized R_0_ extrapolated back to the start of the epidemic, for any particular variant, is of little practical use. It is still the instantaneous R_*t*_ that reflects the situation for the current variant.

## Testing rates

Clearly, increasing the number of people tested will increase the number of positive cases, except when infection has stopped completely. Inevitably, the growth and decay of positive daily cases registered include any changes in the rate of testing.

**Fig. 8:**
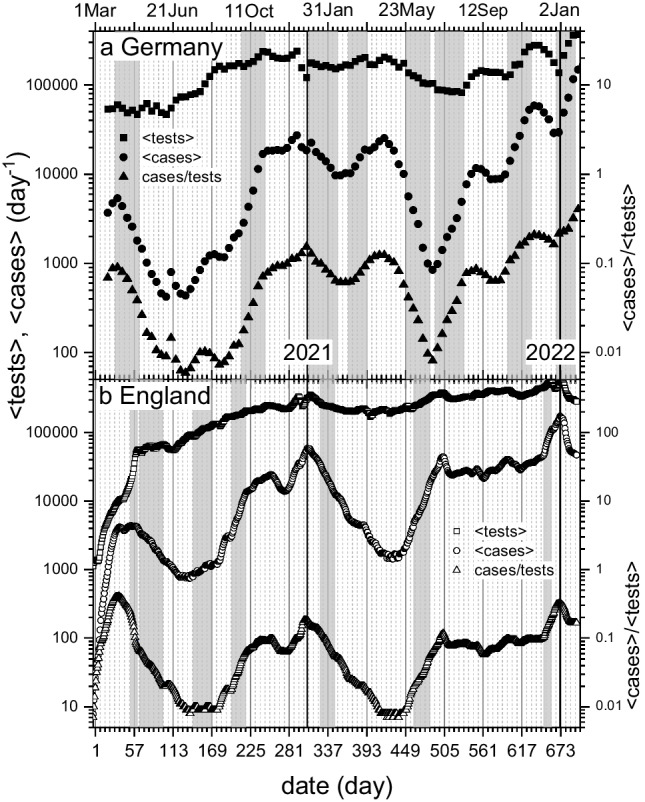
Testing rate, 2020–2022. **a**) Number of tests reported in Germany (${N}_{test}$, squares, left-hand scale), number of positive tests (${C}_{t}$, circles, left-hand scale), and ratio of positive cases to tests reported (${C}_{t}/{N}_{test}$, triangles, right-hand scale). Daily 7-day averages calculated from weekly totals assigned to Thursdays of week reported to RKI (RKI 2021c; 2023b). **b**) 7-day average number of: daily tests in England (squares), and positive tests calculated from 7-day average positivity (circles); 7-day average of cases divided by that of tests (triangles). *x*-axis: day of sample-taking, plotted as centre of 7-day averaging window (UK-Gov. 2022a). *y*-axis is logarithmic; grey-shaded areas are common regions of exponential change

In analogy with compartment population densities [*S*], [*I*] and [*R*] used in Eqs. 1–3, we use the *fraction* of positive cases ${C}_{t}/{N}_{test}$ to allow for changes in testing levels. (Tacitly, this assumes the effective size of population surveyed in a testing regime is proportional to ${N}_{test}$.) Using this normalization, the day-to-day rate of change in number of daily positive cases ${C}_{t}$ is:

21 $$\frac{d({C}_{t}/{N}_{test})}{dt}=\frac{1}{{N}_{test}}\frac{d{C}_{t}}{dt}-\frac{{C}_{t}}{{N}_{test}^{2}}\frac{d{N}_{test}}{dt}={r}_{I}\left(\frac{{C}_{t}}{{N}_{test}}\right)$$

where the second equality defines the rate constant ${r}_{I}$ for fraction of positive cases. In regions of exponential change (i.e., fixed *r*), the rate constant for new daily infections corrected for variable testing thus becomes:

22 $${r}_{I}=r-{r}_{N}$$

where *r* is the usual rate constant for daily cases, and ${r}_{N}={N}_{test}^{-1}d{N}_{test}/dt$ is the effective rate constant for ${N}_{test}$. (The latter is approximated as an exponential change; see Fig. 8 below.)

Figures 8a, b compare numbers of tests, positive cases, and their ratio, for Germany and England, respectively. Numbers are per day, calculated from weekly values for Germany, but from daily averages over a 7-day window for England. Data correspond to official reporting dates for Germany, but relate to the sample date (unlike in Fig. 4c) for England. In both cases, the date refers to the middle of the 7-day period.

The testing data for Germany is that reported to the Robert-Koch Institute (Fig. 8a; RKI 2021c; 2023b). Reporting was voluntary and it took some time to establish a stable number of contributing laboratories. Plotted are: total number of tests reported ${N}_{test}$ (squares); number of tests found positive (circles), and fraction of positive tests relative to reported tests (triangles). Regions where exponential changes in positive cases correspond with approximately exponential change in number of tests are shaded grey in the log-linear plot. From the case data, we see that these correspond consecutively to: (i) Decline of the primary wave. (ii) Growth of the seasonal autumn wave in 2020. (iii) Decrease of the winter wave in 2021. (iv) Growth of the wave associated with the Alpha-variant. (v) Descent into the summer trough. (vi) Growth of the 2021 autumn wave. (vii) Growth of the Delta wave. (viii) Beginning of the Omicron wave. In each of these regions, the fraction of positive tests, ${C}_{t}/{N}_{test}$, also changes exponentially.

For England, case data in Fig. 8b exhibit apparent doubling of the initial peak with extensive broadening in the tail, relative to case data in Fig. 4c. This relates, at least in part, to procedural changes introduced on 2 Jul 2020 (day-124) that we mentioned previously (UK-Gov. 2025). Excepting the first narrow grey band, the regions of common exponential change in Fig. 8b correspond to: (i) Decline of the primary wave; (ii) Summer trough; (iii) Growth of the 2020 autumn wave; (iv) Decrease of the Alpha wave from its peak at the start of 2021.; (v) Growth of the Delta wave; and (vi) Beginning of the Omicron wave. Note the very different locations of the Alpha and Delta waves, relative to Germany, as found already in Fig. 7.

Initially, testing in England was rather low and restricted to the essential Pillar 1, which includes mainly in-hospital tests of patients, and of health and social workers. Testing of the general public, Pillar 2, started somewhat later. Data in Fig. 8b include both Pillars 1 and 2. The anomalously steep rise in the testing rate seen on 1 May, and above (thin grey band), occurs close to a target date that was pre-announced for the end of April. It produces the second bump in positive cases and obscures the sharp decline in the case/test ratio. Beyond this, testing increases more or less steadily with time, on a log-linear scale, up to the end of 2020.The increase in cases at the bottom of the 2020 summer trough reflects entirely this trend in testing. During most of 2021, the testing rate remains much more constant over long periods, and the profile of case numbers reflects better that of the case/test ratio. Once testing in England was up to speed, numbers in Fig. 8b should reflect all tests under the supervision of the (now defunct) Public Health England; unlike the voluntary, and to some extent variable, reporting of tests to the RKI in Germany.

Table 3 compares rate constants for infection ${r}_{I}$, testing ${r}_{N}$, and fraction of positive tests *r*, in the three countries. Values of ${r}_{I}$ agree with the prediction for exponential rates given by Eq. 22. In Germany, the rate constant associated with testing ${r}_{N}$ is 5 − 33% of that for positive cases *r*, with mean $\left|{r}_{N}/r\right|=$ 19 ± 5%. We therefore expect true changes in incidence to account for most of the rate changes found in Figs. 3a, 4b and 10b (given later). The low numbers of tests initially in England cause a dramatically high $\left|{r}_{N}/r\right|$ ratio. Here, the increase in cases is dominated completely by increased testing, as described already. Beyond the 2020 summer trough, the mean $\left|{r}_{N}/r\right|=$ 18 ± 2% reflects stable testing. Data for Italy is less extensive, but also reflects a relatively stable situation. After the time span covered by Fig. 8, changes in rate of testing increase, and both rates of incidence and absolute numbers of cases are likely distorted by insufficient testing, as the pandemic begins to decline.

**Table 3:** Rate constants for new infections ${r}_{I}$, testing ${r}_{N}$ and positive tests *r* (cf. Equation 22) in Germany, England and Italy^a^

range (day)	< case >, *r* (day^−1^)	< test >, ${r}_{N}$ (day^−1^)	< case >/< test >, ${r}_{I}$ (day^−1^)	$\left|{r}_{N}/r\right|$ (%)
*Germany:*				
39‒60	‒0.042 ± 0.003	‒0.006 ± 0.004	‒0.036 ± 0.002	21 ± 6
214‒242 (207‒235)^b^	0.065 ± 0.002	0.014 ± 0.001	0.053 ± 0.002	22 ± 4
312‒347	‒0.023 ± 0.001	‒0.004 ± 0.001	‒0.019 ± 0.001	17 ± 5
368‒389	0.030 ± 0.001	0.010 ± 0.002	‒0.020 ± 0.003	33 ± 8
431‒466	‒0.056 ± 0.003	‒0.012 ± 0.003	‒0.044 ± 0.005	17 ± 3
494‒529	0.044 ± 0.002	‒0.002 ± 0.0004	0.046 ± 0.002	5 ± 2
*England:*				
54‒64	‒0.002 ± 0.001	0.123 ± 0.005	‒0.125 ± 0.005	6150 ± 3325
68‒101	− 0.028 ± 0.001	0.006 ± 0.001	− 0.033 ± 0.001	21 ± 4
145‒171	0.015 ± 0.001	0.014 ± 0.001	0.001 ± 0.001	93 ± 13
201‒221	0.070 ± 0.001	0.008 ± 0.001	0.062 ± 0.001	11 ± 2
329‒350	‒0.046 ± 0.0005	‒0.007 ± 0.001	‒0.039 ± 0.0005	15 ± 2
464‒487	0.055 ± 0.002	0.012 ± 0.0004	0.043 ± 0.002	22 ± 2
652‒662	0.080 ± 0.007	0.020 ± 0.001	0.060 ± 0.002	24 ± 3
*Italy:*				
47‒103	‒0.041 ± 0.003	0.014 ± 0.002	‒0.055 ± 0.001	33 ± 3
222‒271	0.073 ± 0.005	0.019 ± 0.001	0.054 ± 0.004	26 ± 3
453‒502	‒0.050 ± 0.001	‒0.010 ± 0.002	‒0.040 ± 0.002	20 ± 3
509‒530	0.084 ± 0.006	0.009 ± 0.001	0.075 ± 0.005	10 ± 5

^a^From log-linear regressions as in Fig. 8

^b^For case/test

Note that we can get a rough indication of *N*, the total effective population involved in the recorded cases, by comparing with the maximum in infectious population, ${I}_{max}\equiv {[I]}_{max}N$, that Eq. 18 predicts for the SIR model. The infectious population on day-*n* is the sum over all cases, *C*_*i*_, not yet recovered/removed: ${I}_{n}={\sum }_{i=n-{T}_{I}+1}^{n}{C}_{i}$. Here ${T}_{I}(=1/\gamma )$ is the infectious lifetime, i.e., recovery time, which must be at least as long as the serial interval; we assume ${T}_{I}=8$ days (cf. SI-values quoted previously). Using augmented onset-of-symptoms case data (RKI 2021a), we then get ${I}_{max}=39060$ at day-22 for the initial outbreak, and R_0_ = 3.18 from the initial exponential rate (${r}_{0}=$ 0.273 ± 0.009 day^−1^) with Eq. 10, leading to ${[I]}_{max}=0.322$ from Eq. 18 and thus giving *N* = 121,200 for the effective population sampled. This effective *N* is less than but approaches 0.18% of the true population, which is the proportion estimated earlier for Germany by Linka et al. (2020b). Of course, this value of ${I}_{max}$ (and hence *N*) is an underestimate because preventative measures were already introduced by this time.

## COVID-associated deaths

Data on fatalities following diagnosis of COVID, unlike case figures in the previous sections, do not suffer from possible under reporting. However, the definition of COVID-associated death is unlikely to be uniform between countries. In not all cases is COVID necessarily the primary cause of death. Using excess deaths needs a baseline, which preventative interventions may depress below usual levels. Criteria were changed more than once in England, perhaps not surprisingly in a rapidly developing situation.

**Fig. 9:**
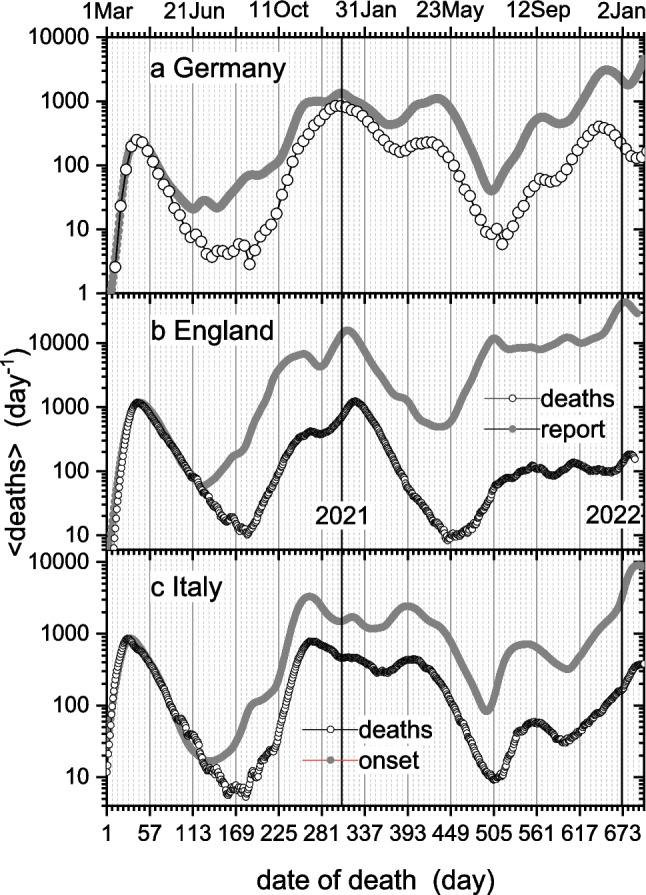
Daily COVID-associated deaths (open circles) for: **a**) Germany (RKI 2021d; and see RKI 2025), **b**) England (UK-Gov 2022b), **c**) Italy (ISS 2025). For Germany, daily deaths are weekly averages centred on Wednesdays. Solid circles: predictions from daily infections as function of symptom-onset date ${t}_{os}$ (Eq. 23), or of reporting date for England. Predictions are scaled to the first peak. Case data: Germany (RKI 2023c), England (UK-Gov. 2021b), Italy (ISS 2025). Probability density function in Eq. 23 is from Fig. 5 (Verity et al. 2020). Day-1 is 1 Mar 2020; *y*-axis is logarithmic

Figures 9a, b and c give the time course of COVID-associated deaths. Open circles are daily values in Germany, England, and Italy, respectively. For Germany, daily numbers come from weekly averages centered on Wednesdays; otherwise, values are 7-day moving averages. Table 2 compares exponential rate constants *r*_*t*_ deduced from regressions in the linear regions of Fig. 9 with corresponding values from the incidence profiles in Fig. 4. In most cases, the two sets of values are quite close. Values of *r*_4_ for deaths are missing from Table 2, because we find no increase of the open circles in Fig. 9 that could correspond to annual summer holidays.

Solid grey circles in Fig. 9 are predictions of daily deaths from the dependence of daily cases ${C}_{{t}_{os}}\equiv -d{S}_{onset}/dt$ on symptom-onset date ${t}_{os}$ (cf. Figure 4b). The number ${d}_{t}$ of deaths on day *t* is the sum over cases with symptoms-onset on previous days ${t}_{os}$, weighted by the probability ${f}_{t-{t}_{os}}^{(os-d)}$ that death occurs $\tau =t-{t}_{os}$ days after onset of symptoms (Flaxman et al. 2020):

23 $${d}_{t}={\left(cfr\right)}_{t}\sum_{{t}_{os}=0}^{t-1}{f}_{t-{t}_{os}}^{(os-d)}{C}_{{t}_{os}}$$

where ${\left(cfr\right)}_{t}$ is the daily case-fatality rate (i.e., ratio of deaths to positive cases). Verity et al. (2020) fit early data for ${f}^{(os-d)}(\tau )$, from Hubei province and elsewhere in China, with a gamma distribution (Eq. 11) yielding mean onset-to-death $\overline{\tau }=$ 17.8 days and standard deviation 8.0 days. Predictions in Fig. 9 depend not only on daily values of ${\left(cfr\right)}_{t}$, but also on degree of completeness of the onset data. For purposes of comparison, we therefore normalize to the first peak in daily number of deaths. With a reduced SD of 6 days and a forward shift of 8 days, the probability density distribution from Verity et al. (2020) describes the shape of the first peak of the epidemic in Germany reasonably well. This is also true for Italy and England, except that the prediction stays unshifted for Italy, and is shifted backward by − 9 days for England. Note that onset data for Germany are augmented by imputation to include missing data. Incidence data for England are based on reporting date, which is shifted up from symptoms onset. Also, as remarked already, testing started rather late in England. (The abrupt jump in reporting data for England in Fig. 4c is removed in Fig. 9 to extend the range useable for comparison – see UK-Gov., 2025.)

After the first-wave peak, predictions are consistently higher than deaths. This means that the case-fatality rate ${\left(cfr\right)}_{t}$ must be smaller, which implies a higher rate of testing than at the first peak (cf. Figure 8). Values of ${\left(cfr\right)}_{t}$ are lowest at troughs in incidence, because then testing captures a larger proportion of positive cases. For Germany (Fig. 9a), the mean value in the first trough between days 130 and 221 is $\overline{{(cfr)}_{t}}=0.0070$ (SD = 0.0024). At the peaks of the first two waves ${\left(cfr\right)}_{t}$ is much higher. If we attribute this solely to under-reporting, then approximately 3/4 and 2/3 of cases escaped reporting at the peaks of first and second waves, respectively (Marsh 2025a). This agrees with Fiedler et al. (2021), who estimate that 74% of cases go undetected at the first peak in Germany. For Italy, in the first trough at days 172–238 of Fig. 9c the case-fatality rate is $\overline{{(cfr)}_{t}}=$ 0.024 (SD = 0.005), and correspondingly for England at days 190–229: $\overline{{(cfr)}_{t}}=$ 0.024 (SD = 0.002).

To compare with these results, in the later first-wave peak around day-47, Fiedler et al. (2021) derive a *cfr* corrected for under-reporting of 0.98% in Germany, and correspondingly of 1.51% in Italy. Verity et al. (2020) find a *cfr* of 1.38% [CI: 1.23–1.53] for China, and the delay-adjusted *cfr* for testing-intensive S. Korea is 1.97% [CI: 1.94–2.00] (Shim 2021). Dimpfl et al. (2021) determine infection-fatality rate *ifr* = 0.86% [CI: 0.69–0.98] for Germany. Additionally, linking COVID-associated deaths in 45 countries with 22 national-level surveys of seroprevalence yields a weighted average *ifr* = 0.96% (O’Driscoll et al. 2021). Of course, *cfr* exceeds *ifr* whenever some positive cases go unreported.

Beyond the timescale shown in Fig. 9, peak numbers of deaths from dominant Omicron variants BA.2, BA.5 and BF.7 decline throughout 2022 and into 2023, following intensive programmes of vaccination. Over the range days 704–935, the mean value of ${\left(cfr\right)}_{t}$ for Germany reduces to $\overline{{(cfr)}_{t}}=0.0013$ (SD = 0.0003), and that over days 674–1002 in Italy becomes $\overline{{(cfr)}_{t}}=0.0073$ (SD = 0.0014). At least part of the large decrease comes from all preventive measures introduced by this stage. The reduced pathogenicity of Omicron variants is a further cause for the decrease. This was evident also in the hospitals, and is an important global contributor to recovery from the pandemic.

## Prognoses

In the absence of interventions, final outcomes depend on the basic reproduction number ${\mathrm{R}}_{0}$ that we determine at the beginning of the epidemic. If we describe the emerging outbreak as a branching process, the balance equation (Eq. 19) gives the probability $\Pi$ that a major outbreak develops. Taking $m=2.25$ for the exponent in Eq. 19 (Bi et al. 2020), the probabilities ${\Pi }_{0}$ of major outbreaks in Germany, Italy and England become those listed in Table 4. We can estimate the final fraction ${\rho }_{0}$ of infections that ensues from a major outbreak by using the final-size equation, i.e., Eq. 17. These values also are included in Table 4. Because Eq. 17 applies for a gamma distribution of infective lifetimes (Ma and Earn 2006), we retain the same ${\mathrm{R}}_{0}$ for this comparison.

**Table 4:** Basic reproduction number ${\mathrm{R}}_{0}$, initial probability of major outbreak ${\Pi }_{0}$, final fraction of infections ${\rho }_{0}$, and subsequent changes, ${\mathrm{R}}_{t,5}$, ${\Pi }_{5}$, ${\rho }_{5}$, for Germany, Italy and England^a^

country	${\mathrm{R}}_{0}$ ^b^ [range, day]	${\Pi }_{0}$, ${\rho }_{0}$	${\mathrm{R}}_{t,5}$ [range, day]	${\Pi }_{5}$, ${\rho }_{5}$
Germany	3.46 [17‒24]	0.846, 0.964	1.53 [133‒157]	0.455, 0.602
Italy	2.49 [0‒11]	0.740, 0.891	1.70 [136‒164]	0.531, 0.691
England	3.13 [8‒19]	0.819, 0.949	1.48 [134‒163]	0.427, 0.570

^a^From Eq. 19, with *r*_*t*_ from Table 1 (*m* from Bi et al. 2020) for ${\Pi }_{0}$, and correspondingly Eq. 17 for ${\rho }_{0}$

^b^ ${\mathrm{R}}_{0}$ for gamma-distribution (Eq. 12; Bi et al. 2020). Similarly for ${\mathrm{R}}_{t}$

Clearly, the probability of a major COVID-19 outbreak was very high, if preventative steps were not taken promptly. Otherwise, we would expect ultimately that nearly the whole of the population become infected. Italy seems initially to have been slightly better placed, and England to have improved by the end of the first summer. Prognoses are better when ${\mathrm{R}}_{t}$ is reduced closer to unity, but such predictions apply only so long as preventative measures causing a decreased ${\mathrm{R}}_{t}$ remain in place. Lasting effects come, however, from immunity retained after infection, and most importantly from vaccination.

The basic reproduction number ${\mathrm{R}}_{0}$ determines how much of the population we must vaccinate to halt the epidemic. If we remove fraction *p* of susceptible individuals by immunization, the effective reproduction number is reduced to $\mathrm{R}={\mathrm{R}}_{0}(1-p)$. The condition $\mathrm{R}<1$ for steady decay of the disease then leads to the critical proportion of the population that must be vaccinated successfully:

24 $${p}_{c}=1-1/{\mathrm{R}}_{0}$$

Taking ${\mathrm{R}}_{0}$ from Table 4, we get critical values of ${p}_{c}$ = 0.71 ± 0.01, 0.60 ± 0.01 and 0.68 ± 0.01 for Germany, Italy and England, respectively. According to the RKI (2023d), 71% of the population in Germany achieved basic immunity (twice vaccinated, or once vaccinated after pre-infection) by the end of 2021 (day-668). According to the ECDC (2023b), 60% of the population in Italy had the primary course of vaccination by week-34 of 2021 (day-544). Note that vaccination was prioritized for the elderly, because they are most susceptible; and also for critical health workers. But it is coverage of the whole population that is relevant for approaching herd immunity by vaccination.

In Eq. 24, we may need to scale up values of ${\mathrm{R}}_{0}$ from those of the original SARS-CoV-2, when a new variant that is more effective in transmission comes to dominate. This is the case for Alpha. Vaccination levels in Germany were very low at the time of the Alpha wave. Hence, the accompanying low instantaneous ${\mathrm{R}}_{t}=$ 1.24 (see Fig. 6a) came from preventative measures that mostly were impermanent. Using the ratio $R_{t,\alpha}/R_{t,orig}$ from Table 2 as an estimate gives a corrected ${\mathrm{R}}_{0}=4.9$ that increases the critical vaccination fraction to ${p}_{c}$ = 0.80 for the Alpha variant. Corresponding revised values are ${p}_{c}$ = 0.70 for Italy; and 0.83 for England, where Alpha originated.

Later into the vaccination programme, preventative restrictions were lifted and the instantaneous ${\mathrm{R}}_{t}$-levels reflected permanent effects of increased immunity in the community. If ${v}_{t}$ is the fraction of the population fully vaccinated (or otherwise immune) by this time *t*, then the remaining fraction that we should vaccinate is: $(1-{v}_{t})(1-1/{\mathrm{R}}_{t})$. For instance, by onset of the 2021 delta-wave in Germany (day-501), 43% of the population were doubly vaccinated and the instantaneous reproduction number from Fig. 6a was ${\mathrm{R}}_{t}=$ 1.45. The further fraction of (double) vaccinations needed is then 18%, totaling 60% in all, which is less than estimated from the original ${\mathrm{R}}_{0}$. However, this increased back to 72% towards the peak of the Delta outbreak, and further to a target total of 78% on approaching the first Omicron peak. In Italy, ${\mathrm{R}}_{t}$ for Omicron BA.1 was higher than for preceding variants (see Fig. 6b). The revised total immunization needed at the beginning of the Omicron wave then reaches 89%, much exceeding the estimate made above for the start of the Alpha wave.

## Conclusion

**Fig. 10:**
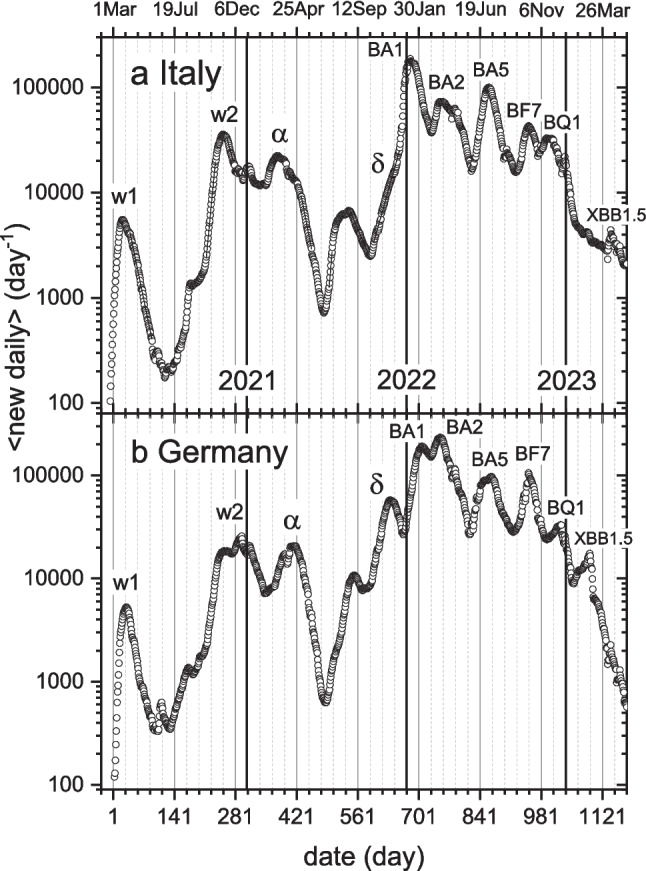
Daily number of new cases over the entire epidemic. 7-day averages according to: **a**) sample/diagnosis date for Italy (ISS 2025); **b**) reporting date for Germany (RKI 2023c). Infection waves (w1, w2, …), including those associated with COVID-variants (α, δ, BA1, …) are labeled. Day-1 is 1 Mar 2020; *y*-axis is logarithmic

There are many, and disparate, things to consider. Therefore, I number them (rather arbitrarily):

1. To put all aspects reviewed into perspective, Figs. 10a, b show the full profile of daily cases over the period from March 2020 to May 2023, for Italy and Germany, respectively. At the beginning of May 2023, the WHO officially announced the end of the pandemic (day-1161). We see that by this time daily case numbers have fallen to a level far lower than in preceding Omicron waves. The XBB.1.5 subvariant remained at low levels in Germany until the end of the summer in 2023, and only then was it replaced during the much-attenuated autumn/winter wave.

The first two waves in Figs. 10a, b are the initial outbreak w1, and the first seasonal autumn/winter wave w2. Next follows the wave from the Alpha variant. Subsequent waves coincide with the appearance of succeeding new dominant variants. Seasonality does not disappear, however; it drives the summer troughs, prominently that in 2021. Winter seasonality augments the original Alpha wave in England, relative to Italy and Germany (see Figs. 6, 8, 9), and correspondingly autumn seasonality augments the Delta wave in Italy and Germany. Winter seasonality heightens the first Omicron wave for all three countries.

2. Representing incidence by daily case numbers, as done here, involves quite some uncertainties. It is therefore encouraging that the amplitude of weekly modulation remains constant up to day 297, in Germany (Fig. 3b). A further positive aspect is that by concentrating on exponential rates *r* we automatically achieve an internal scaling, because the rates come (via the logarithm) from ratios of daily cases.

Initial r﻿ates, ${r}_{0}$, are particularly important as they give us the basic reproduction number, ${\mathrm{R}}_{0}$. Because case numbers are low initially, as are rates of testing, variability is likely to be high (see Fig. 6). Mercer et al. (2010) propose that conditions prevailing at the beginning of outbreaks intrinsically can lead to overestimating ${\mathrm{R}}_{0}$. It is therefore significant that ${r}_{0}$ from rates of COVID-associated fatalities mostly agrees with estimates from cases (Table 1).

3. Much relies on distribution functions for generation times and other time intervals, particularly to determine reproduction numbers. We take these distributions as given in the calculations performed here. Error estimates are based solely on standard errors of exponential fits to the time series. Uncertainties in the distributions are not included. In principle, we can convert credible-interval (CI) ranges for distributions to standard errors by assuming Gaussian distributions. This would facilitate incorporation into the error scheme used here. In the absence of this, we afford a limited sensitivity analysis by comparing results obtained with variously determined different distributions.

A serious and perennial issue is use of (relatively widely available) serial intervals, instead of (essentially inaccessible) generation times. Serial intervals are based on onsets of symptoms, which are observable, but generation times refer to the points of infection, which are observable neither for infector nor infectee, and only can be inferred indirectly. This problem is particularly thorny for COVID, because of presymptomatic transmission (infectious before onset of symptoms) that causes SIs to extend to negative values, which generation times cannot. The latter complicates choice of lower limits for the integrals involved, as discussed already in connection with Eq. 13, and in the results section on ${\mathrm{R}}_{0}$ (Marsh 2025b). Generation times are determined only indirectly, by modelling or statistically, with results depending on the methods used (see e.g., Hart et al. 2021; Hart et al. 2022; Ganyani et al. 2020). As said already in the previous paragraph, with SIs we can compare a range of independent determinations.

4. Unsurprisingly, we get different values of ${\mathrm{R}}_{0}$ depending on choice of SI distribution and its parameters. This is part of a wider range of variability in ${\mathrm{R}}_{0}$-determinations depending on the methods used. Brockhaus et al. (2023) identify various factors causing the spread of values obtained from different protocols for tracking the instantaneous ${\mathrm{R}}_{t}$ in Germany. In addition to those found here, they conclude that many other analytical choices contribute to variability, at least as much as does the choice of statistical method. Note that ${\mathrm{R}}_{t}$-values in Fig. 6 use SI-data from early in the outbreak, and do not allow for decreases due to preventative measures and dynamics of the pandemic itself (Ali et al. 2020; Park et al. 2021). This would tend further to decrease peaks in ${\mathrm{R}}_{t}$, after the initial one.

5. For daily positive cases to reflect incidence reliably, we need sufficient testing. Also, the rate of testing must be stable. At early stages in England, daily cases were dominated by rapid increases in the testing rate (Fig. 8b). Of course, more tests are always desirable, because then more cases can be identified and dealt with. But we must recognise possible distorting effects on case rates, which is especially critical for the authorities responsible who rely on these data. Ultimately, England achieved a high and stable rate of testing (Fig. 8b). Possibly one of the highest, but again it is difficult to compare directly between countries. As mentioned already, reporting numbers of tests in Germany was voluntary. Laboratory-based PCR testing is the standard used for data collection. Unrecorded home test kits are less sensitive, and hence prone to false positives, but nonetheless they are important for self-isolation.

6. COVID-associated deaths track the severe part of daily incidence, and afford approximate estimates of under-reporting. Initially, deaths were high in England and Italy (Figs. 9b, c), but as noted already, definitions and protocols differ between countries. For instance, deaths recorded 28 days after diagnosis in England were lower than medical data recorded with the death certificate that we use in Fig. 9b. Hospital admissions and occupancy of intensive-care units also follow daily incidence in a similar manner. The latter are absolutely crucial to pandemic management and ensuring that essential health services are not overwhelmed. Other data sources relevant to infection levels include seroprevalence of antibodies, and viral load in waste water.

7. COVID vaccines have different efficacies, *e*, depending also on the current viral variant. Values vary from 95% for mRNA vaccines to 50% for inactivated virus (Mahase 2021). Vaccine coverage estimated from Eq. 24 in the previous section assumes a perfect vaccine with *e* = 1. For all-or-none efficacy (where fraction *e* of those vaccinated becomes completely immune and the remainder receive no protection), the critical fraction ${v}_{c}$ of the population that we must vaccinate increases such that: ${p}_{c}=e{v}_{c}$, where ${p}_{c}$ is given by Eq. 24 (Smith et al. 1984). At the opposite extreme (the “leaky” vaccine), everyone vaccinated has chance 1 − *e* of infection on encountering an infector. For the first round of encounters, fraction 1 − *e* become infected, the same as for all-or-none. However, those evading infection on the first round still have probability 1 − *e* of infection on the second and following rounds, whereas remaining all-or-none vaccinees by now are all immune (Smith et al. 1984). For less efficacious vaccines, we should factor this in, as suggested already for changes in ${\mathrm{R}}_{t}$.

8. We assume a homogeneous population here, throughout. Amongst relevant inhomogeneities are age and geographical location. Both are included in COVID epidemiological databases. Indeed, specific inhomogeneities can serve as labels to distinguish different statistical models. For instance, age profiles may link statistical variables such as infection prevalence and fatality, and link across different countries (see O’Driscoll et al. 2021). Interestingly, reproduction numbers for the first wave in Germany were highest for 35–59-year-olds and above, not just for the elderly with their weaker immune response (Marsh 2025a).

9. Some online data used is no longer available on the original websites. Similarly, weekly or monthly summaries may now have replaced the contemporary daily data. For reasons of speed and efficiency, there also is an increasing tendency to present data according to recording date. This is even the case for COVID fatalities, where the date of death would straightforwardly remove an important source of uncertainty.

Of course, it is impossible for me to attempt answers to all, or even any, of these problems. Nevertheless, I hope that my review offers some small help in this direction, particularly from the viewpoint of biophysicists. But above all, I hope that this is a suitable tribute to Ian.                                                      

## Data Availability

No datasets were generated or analysed during the current study.

## Declarations

None is applicable.
